# Annual Meeting of Southern Medical Association to Be Held in Dallas in 1915

**Published:** 1914-12

**Authors:** 


### Medical Society Notes.

THE meeting of the North Texas Medical Association will be held in Dallas on December 8th and 9th. We regret that the committee failed to send us a program, but we feel it will be interesting and profitable. We know the caliber of the North Texas doctors. Be sure and go if you can.

---

ANNUAL MEETING OF SOUTHERN MEDICAL ASSOCIATION TO BE HELD IN DALLAS IN 1915.—The Southern Medical Association, in convention at Richmond, Va., voted to hold the 1915 convention in Dallas. The Association embraces sixteen Southern States. The average attendance at the annual convention is 4000. It is expected to be the most important convention held in Dallas in 1915.

Five other cities were candidates for the 1915 convention. Dallas was placed in nomination before the board of counsellors by Dr. Frank G. Boyd, of Fort Worth, and the nomination was seconded by Dr. E. H. Cary, of Dallas.

Dr. Oscar Dowling, of New Orleans, was elected President of the Association for 1915.

